# Prosit Transformer: A transformer for Prediction of MS2 Spectrum Intensities

**DOI:** 10.1021/acs.jproteome.1c00870

**Published:** 2022-04-12

**Authors:** Markus Ekvall, Patrick Truong, Wassim Gabriel, Mathias Wilhelm, Lukas Käll

**Affiliations:** † Science for Life Laboratory, School of Engineering Sciences in Chemistry, Biotechnology and Health, Royal Institute of Technology—KTH, Box 1031, SE-17121 Solna, Sweden; ‡ Computational Mass Spectrometry, Technical University of Munich (TUM), D-85354 Freising, Germany

**Keywords:** Machine Learning, Proteomics, MS2 Spectra, Transformers

## Abstract

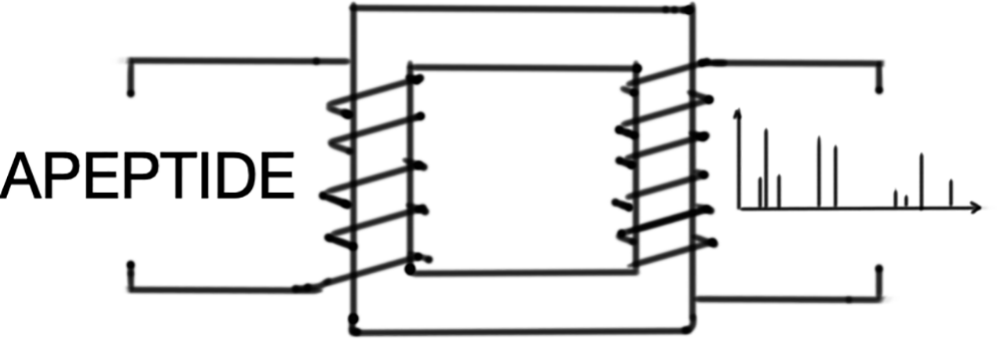

Machine learning has been an integral part of interpreting data from mass spectrometry (MS)-based proteomics for a long time. Relatively recently, a machine-learning structure appeared successful in other areas of bioinformatics, Transformers. Furthermore, the implementation of Transformers within bioinformatics has become relatively convenient due to transfer learning, i.e., adapting a network trained for other tasks to new functionality. Transfer learning makes these relatively large networks more accessible as it generally requires less data, and the training time improves substantially. We implemented a Transformer based on the pretrained model TAPE to predict MS2 intensities. TAPE is a general model trained to predict missing residues from protein sequences. Despite being trained for a different task, we could modify its behavior by adding a prediction head at the end of the TAPE model and fine-tune it using the spectrum intensity from the training set to the well-known predictor Prosit. We demonstrate that the predictor, which we call Prosit Transformer, outperforms the recurrent neural-network-based predictor Prosit, increasing the median angular similarity on its hold-out set from 0.908 to 0.929. We believe that Transformers will significantly increase prediction accuracy for other types of predictions within MS-based proteomics.

## Introduction

Just as in many other areas involving the analysis of large and complex data sets, different types of machine learning are tremendously helpful for the modern analysis of mass spectrometry (MS)-based proteomics data.^1,2^ For example, we nowadays can use machine learning to predict tryptic digestion,^3^ chromatographic retention time,^4−6^ collisional cross section,^7^ the accuracy of peptide–spectrum matches,^8^ and the accuracy of transitions in DIA data^9^ are tasks that utilize machine learning.

One task that has gained traction in the last couple of years is predicting MS2 spectra from peptide sequences.^10,11^ Such predictors can predict relative intensities of a given peptide sequence’s *b*- and *y*-ions. Together with the *m*/*z* values of the ions, which one can derive from first principles, one can subsequently form a full MS2 spectrum. MS2 spectrum prediction has in a short time established itself as a means to rescore peptide spectrum matches,^12^ increasing the sensitivity in large search spaces,^13^ and target–decoy strategies for DIA interpretation.^14^

Many types of frameworks are available for training a predictor, such as support vector machines and recurrent neural networks (RNNs) used within MS-based proteomics. However, in the last couple of years, a structure first in natural language processing^15^ known as Transformers^16^ has successfully been employed within bioinformatics, e.g., structure prediction,^17,18^ gene expression prediction,^19^ and even within MS-based proteomics, e.g., peptide detection problem,^20^ DIA library generation for the phosphoproteome,^21^ and de novo interpretation of MS2 spectra.^22^

Transformers are, like RNNs, designed to handle sequential input data and do so through attention mechanisms, i.e., mechanisms that enhance the essential parts of the input sequence for its output. However, unlike RNNs, the Transformers do not use recurrence, thus enabling a significant speed-up by parallelizing their training. The encoder–decoder structure is the basis of the Transformers, where both the encoder and decoder adopt the multiheaded attention mechanism.^16^

Notably, the task assessing protein embedding (TAPE) model^17^ is exciting; a Transformer-based autoencoder of protein sequences is formed by withholding one amino acid at a time in a large set of protein sequences and subsequently predicting which is the missing amino acid. One can subsequently employ the model for higher-level tasks by plugging them into some extra layers of neurons in a process known as transfer learning.^17,18^

Here, we argue that Transformers can greatly aid MS-based proteomics. We demonstrate that TAPE’s BERT submodel can predict MS2 spectrum intensities from peptide sequences. We are using the training and test sets of the popular Prosit^11^ predictor and demonstrate that the transformer-based predictor, which we named Prosit Transformer, drastically outperforms the old implementation of Prosit.

## Methods

### Data

We downloaded the Prosit training data from https://figshare.com/projects/Prosit/35582. This set is composed of spectra from PXD004732, PXD010595, and PXD021013.^11,13^ The Prosit data had to be converted from HDF5 to LMDB to be compatible with the TAPE framework. The LMDB data files used during training and validation are accessible at https://figshare.com/articles/dataset/LMDB_data_Tape_Input_Files/16688905.

### Architecture

The TAPE model consists of 12 768 hidden unit attention layers, with the attention dropout (DropHead) rate^23^ and regular dropout rate set to 0.1. We downloaded weights for the pretrained model that has been trained on the raw protein sequences in the protein families database (Pfam) to predict the amino acid at each protein position given the previous amino acids and the following amino acids.^17^ The Prosit-specific transformer has the same parameter but consists of nine attention layers. The metadata layer is a multilayer perceptron (MLP) with two layers of size 512 units followed by a dropout rate of 0.1 each. The final prediction layer has the same structure, except for no dropout after the final layer. The activation function is ReLU, except for the prediction layer where the first layer uses a ReLU6,^24^ i.e., a max(0,min(6,*x*)) function as an activation function, and the final layer uses a linear layer.

### Metrics

We measure angular distance
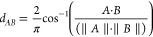
and angular similarity, *s*_*AB*_ = 1*d*_*AB*_ as measures of accuracy of the predicted intensities. Here, *A* is the vector of predicted intensities, and *B* is the vector of observed intensities for the ion series included in the prediction. However, we introduced a few extra steps during training to avoid undefined behavior. First, to avoid undefined values using angular similarity during training, we had to clip the inputs to cos^–1^ with −(1 – ϵ) and (1 – ϵ) to avoid undefined values. This implementation was necessary since some predictions were too similar to their target after training, resulting in an undefined loss. However, there was no clipping during the evaluation, so it will not affect the final result. Lastly, we also had to introduce a small ϵ in the denominator in the cosine similarity, i.e., max(∥*A*∥·∥*B*∥, ϵ), to ensure no undefined behavior during training. The sum of all *d*_*AB*_ for all peptides in the test set was used as a loss function for the training of the networks.

We calculated the FDR = FP/(FP + TP) and FNR = FN/(FN + TP) for each predicted spectrum to measure the number of erroneous peak predictions. Here, FP is the number of peaks predicted in excess to be present in a spectrum that was absent in the observed spectrum; FN is the number of peaks deficiently predicted to be absent in a spectrum that was present in the observed spectrum, and TP is the number of peaks accurately predicted to be present in a spectrum that was present in the observed spectrum.

### Postprocessing of Predicted Intensities

We use the same postprocessing on the predicted spectrum used in Prosit^11^ for the final result. To clarify, we set ions with a predicted negative intensity to zero, i.e., a negative intensity indicates an absent peak. Furthermore, we set all ion’s intensity that is not obtainable for any given peptide due to too low a charge state or too low peptide length to −1. However, we exclude such peaks for similarity measurements.

### Hardware

The model was trained on the Berzelius SuperPOD, a GPU cluster consisting of 60 NVIDIA DGX A100 systems, linked on a 200 Gbit/s NVIDIA Mellanox InfiniBand HDR network.

## Results

We set out to test whether Transformers are a technology fit for spectrum intensity predictions, i.e., to predict the intensities of the most commonly observed ion series (*b*^+^, *b*^2+^, *b*^3+^, *y*^+^, *y*^2+^, and *y*^3+^) of product ion spectra from peptide fragmentation. The length of the peptides ranged between 7 and 30 amino acids long. We used the train/test data and the preprocessing coming with the Prosit predictor as a testbed. Prosit’s scripts calculating the intensity vectors, adopting metadata, and calculating predictions’ angular similarity have been found to be robust after years of use. We also found it straightforward to set up a benchmark, as we could reuse the Prosit test sets just out of the box. We will refer to the traditional Prosit predictor as Prosit RNN from hereon to avoid confusion.

### Model

**Figure 1:**
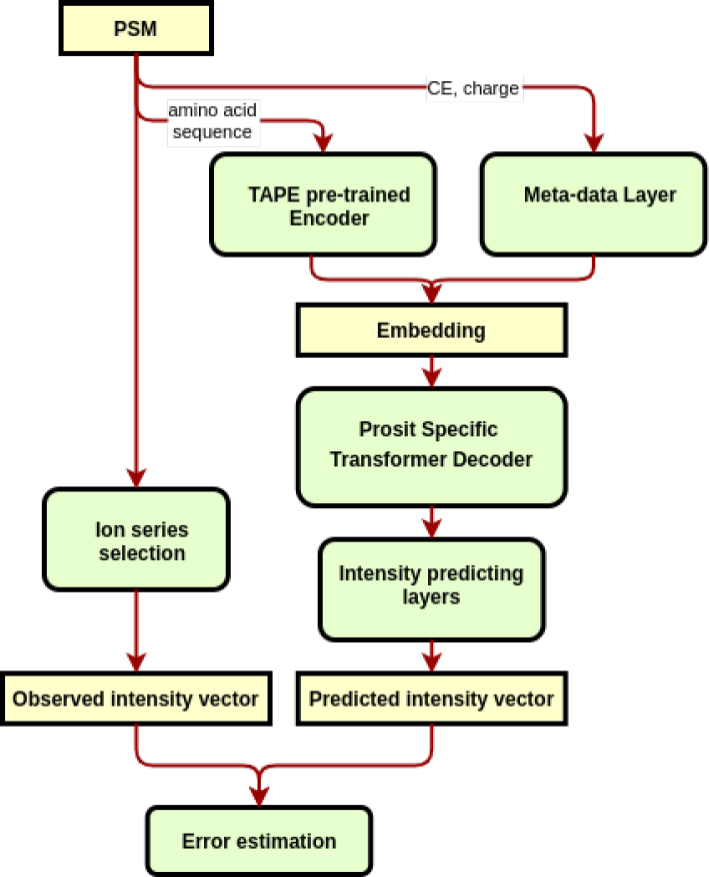
Architecture of the Prosit Transformer. The model depends on a pretrained encoder from the TAPE project and uses the TAPE design for a Prosit-specific decoder. However, our model implements many of the design features of Prosit RNN, i.e., layers handling metadata and final intensity prediction.

We set out to use the setup previously used for training and testing the Prosit model but with a transformer. We used the pretrained TAPE model^17^ and retrofitted it with a Prosit-specific decoder and some additional application-specific code (see Figure 1). The TAPE model will encode the peptide into a 512-dimensional embedding. Furthermore, just as for the original RNN-based Prosit model, we used layers for handling metadata consisting of the charge state of the spectrum and its collision energy (CE). The charge states range from one to six, represented as six-dimensional one-hot encoding. Hence, the metadata layer has seven input nodes to account for the charge state and CE. The metalayer transforms the metadata into a 512-dimensional vector that is subsequently combined with the encoded peptide by element-wise multiplication. Then a Prosit-specific Transformer will decode this combined embedding. Lastly, a two-layered multilayer perception (MLP) follows the decoding layer, serving as a prediction layer to predict the spectrum intensity. The MLP used activation by a hinge loss function constrained between 0 and 6 (a *RELU6* function) to activate the two final layers to avoid a so-called gradient explosion. For the training, the objective function was to minimize the sum of the angular distances between the observed and predicted spectrum intensity vectors.

### Training of the Model

During the training, we used a batch size of 1024, a learning rate of 0.0001, gradient accumulation step of 1, and a linear learning rate schedular with 10000 warmup steps. The training proceeded until no further improvement over 10 epochs.

**Figure 2:**
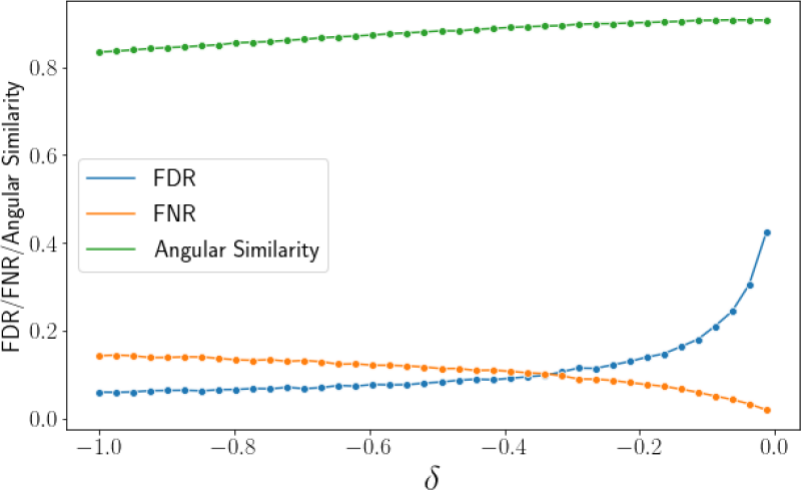
Effect of adjusting the hyperparameter δ on predicting the absence/presence of individual MS2 peaks. To obtain better prediction accuracy of present and absent MS2 peaks, we adjusted the intensities of absent peaks from zero to δ. We measured the false discovery rate (FDR) and the false negative rate (FNR) of each spectrum and then plotted the average angular similarity, the FDR, and the FNR for different choices of δ. We selected δ = 0.34 for the final training. The predicted spectra were not postprocessed for the measurements in this figure (see Methods).

To better predict present and absent peaks, we introduced a hyperparameter, δ, setting an artificial offset of the intensities of absent peaks to δ_p_ = δ/|number of considered peaks|. This hyperparameter adds an extra penalty if the model predicts intensities for absent peaks. By varying the size of δ, we can control the model’s propensity to predict peaks as absent and, by such means, tune the model’s false positive and false negative predictions. We measured the false discovery rate (FDR) and the false negative rate (FNR) of each spectrum and then plotted the average angular similarity, the FDR, and the FNR for different choices of δ. We selected δ = 0.34 for the final training (see Figure 2).

### Comparison of Performance to Regular Prosit

**Figure 3:**
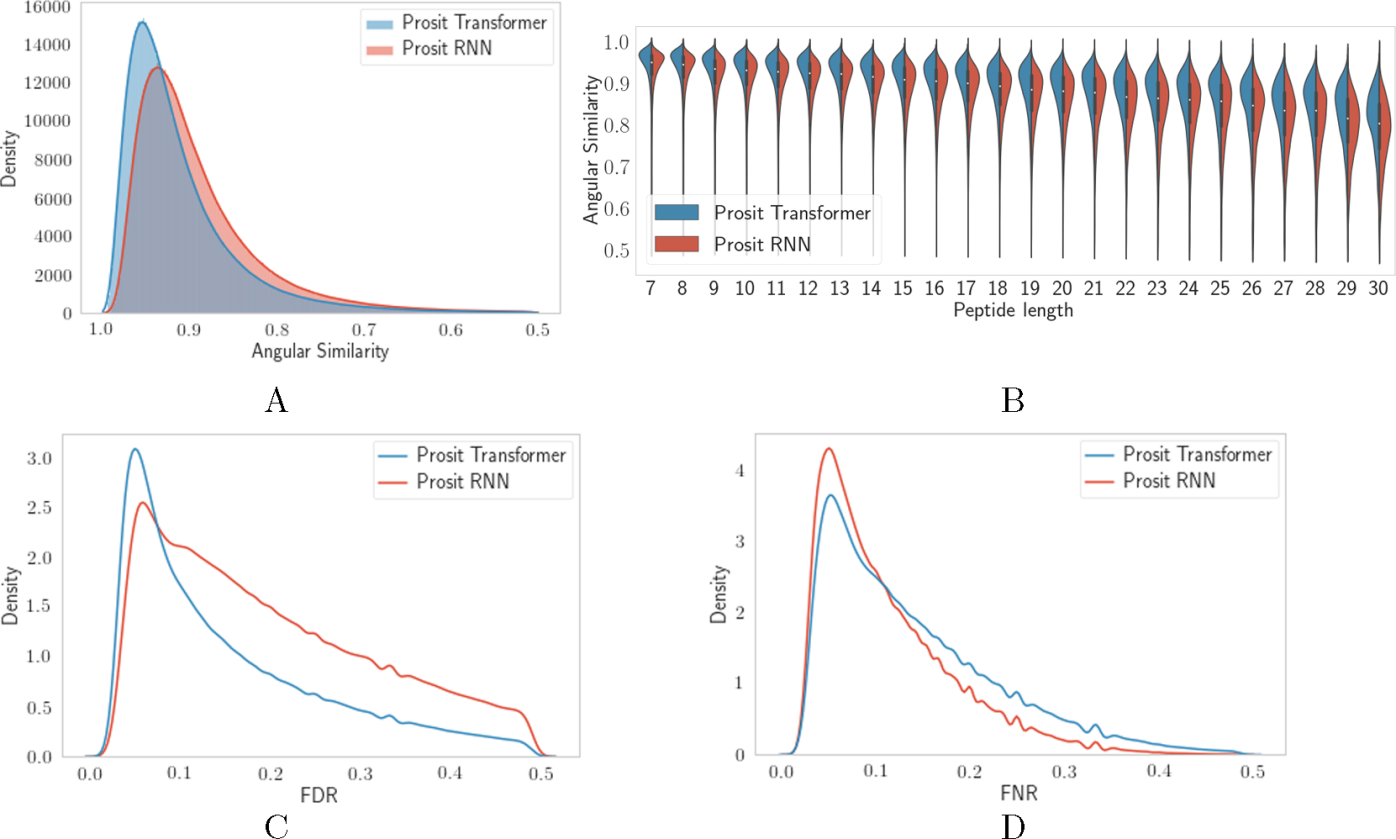
Comparison of the accuracy of Prosit Transformer and Prosit RNN. (A) We made separate histograms and smoothed them with a kernel density estimator to observe the distribution of angular similarity for the spectra predicted with Prosit Transformer and Prosit RNN. (B) Same angular similarity was also stratified by the length of peptides. We also measured the (C) false discovery rate, i.e., the fraction of observed absent peaks among the predicted nonzero intensity peaks for each spectrum, and (D) false negative rate, i.e., the fraction of predicted absent peaks among the observed nonzero intensity peaks.

To test the performance of our final Prosit Transformer, we investigated its performance on the same held-out test set as used when initially training Prosit RNN. We calculated the so-called angular similarity between the predicted and observed intensities for both predictors. Overall, we see that the predictions from Prosit Transformer have an angular similarity higher than that of Prosit RNN and are hence more accurate (Figure 3A). The Prosit Transformer increased the median angular similarity from Prosit RNN’s 0.908 to 0.929. We also see that Prosit Transformer obtained an angular similarity higher than that of Prosit RNN in 75.7% of the spectra, whereas the opposite was true in 24.3% of the spectra. The same pattern was also true when dividing the PSMs based on their peptide’s lengths (Figure 3B). We also wanted to compare the predictors’ ability to predict present and absent (zero intensity) fragment peaks. Our choice of hyperparameter δ for Prosit Transformer resulted in a lower fraction of observed absent peaks among the predicted nonzero intensity peaks (Figure 3C) while observing a higher fraction of predicted absent peaks among the observed nonzero intensity peaks (Figure 3D) for Prosit Transformer compared to Prosit RNN.

### Comparison of a Transformer to an Extended RNN for Prediction of Spectra

We set out to eliminate other explanations for Prosit Transformer’s elevated performance than the Transformers themselves. A notable difference between Prosit RNN and Prosit Transformer is their difference in size. Prosit RNN contains 3 million parameters, while Prosit Transformer contains 164 million parameters, which gives the Transformer an unfair advantage. Hence, we stacked long short-term memory layers to create RNN models of similar size to the ones of the Transformers. This extended RNN gave a median angular similarity of 0.892 compared to Prosit Transformer’s 0.929. Further, Prosit Transformer also outperformed the extended RNNs encoder in combination with Prosit Transformer’s decoder (median angular similarity of 0.927), as well as Prosit Transformer’s encoder in combination with Prosit RNN’s decoder (median angular similarity of 0.915). See Table 1 for an overview of the permutations of encoder decode architectures and their sizes.

**Table 1:** Extended RNN Model’s Size and Performance^a^

architecture for encoder–decoder	encoder size	encoder layers	encoder units	decoder size	decoder layers	decoder units	total size	median angular similarity
Transformer–Transformer	85M	12	768	64M	9	768	164M	0.929
RNN–RNN	77M	5	1028	93M	5	2056	178M	0.892
Transformer–RNN	64M	9	768	94M	10	768	172M	0.9156
RNN–Transformer	53M	6	768	113M	6	768	173M	0.927

a We trained and tested different permutations of expanded RNNs and Transformers of comparable size and compared their prediction accuracy.

When training the RNN models, the learning rate had to be decreased from 0.0001 to 0.00008 to get the model to learn. Everything else was the same as for the Transformer–Transformer model. We also had to switch the gated recurrent unit of the Prosit RNN to an LSTM to use the TAPE framework, leading to minor differences between the extended Prosit RNN and Prosit RNN.

Surprisingly, the extended RNN–RNN model got worse results than regular Prosit. The decrease could be due to that increase from 3 to 178 M parameters, leading to overfitting, requiring more data to justify such a massive model for the type of architecture. However, a performance increase was observed in all cases when adding a Transformer to the architecture. The most significant increase in performance appeared when implementing the Transformer as a decoder, i.e., after the peptide has been encoded and combined with the metadata, and not in the peptide’s encoding, although this improves the results, as well.

At first, the conclusion that the Transformer–Transformer model performed best might seem to contradict the results of others. Particularly, DeepPhospho^21^ reports a better performance for their LSTM–Transformer model than for their Transformer–Transformer model. However, it is worth noting that the circumstances were different; their LSTM decoder was larger than their Transformer decoder (34 M vs 6 M parameters).^21^ One would expect that the Transformer’s performance would increase with a larger model, whereas the LSTM would not benefit as much (perhaps even getting worse) with a larger model.

### Time Comparison of Spectrum Prediction

The Prosit Transformer was quicker to train than the full RNN model (approximately 3 versus 6 GPU days). However, all of the models in Table 1, were slower than the original Prosit RNN due to their increased size. To demonstrate this, both regular Prosit and Prosit Transformer were timed for predicting 1000, 10000, and 100000 spectra; see Table 2. Prosit Transformer requires roughly 40 times more time, so there is a trade-off between accuracy and time requirements for the transformer’s predictions when increasing model size.

**Table 2:** Larger Transformer Model Needs More Time to Predict Spectra^a^

number of predicted spectra	1k	10k	100k
Prosit RNN	0.05 s	0.5 s	4.7 s
Prosit Transformer	2 s	18 s	180 s

a We measured the required time to predict spectra from peptides for both Prosit RNN and Prosit Transformer.

### Prosit Transformer’s Ability to Model Collision Energy

**Figure 4:**
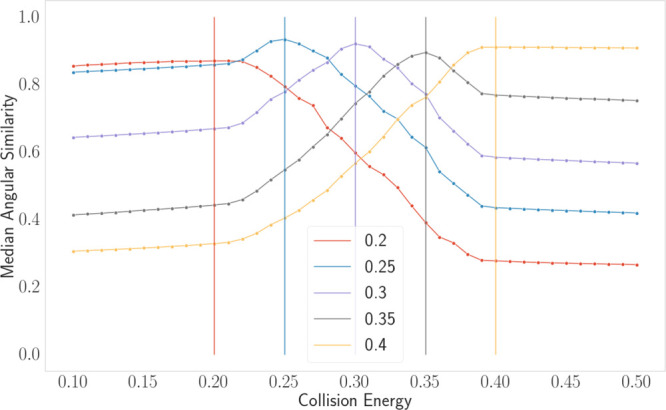
Mean spectral angle as a function of the collision energy for spectra acquired with different CEs.

We also wanted to test that the improved ability of Prosit Transformer to predict MS2 intensities did not affect the predictor’s ability to model CE’s influence on predicted spectra. We hence isolated batches of spectra with CE = {0.2, 0.25, 0.3, 0.35, 0.4} and measured the median angular similarity when predicting the spectra for a range of different collision energies (Figure 4). The highest angular similarity was found between the observed and predicted spectra when setting CE to the set’s actual specified value.

## Discussion

Here, we have used a Transformer trained to predict a protein sequence and transferred its functionality into predicting intensities of the *b*- and *y*-ions of MS2 spectra. The resulting predictor’s performance outperformed a predictor built by a classical recurrent neural network. This type of structure can likely improve other types of peptide property prediction.

One interesting finding was that the most significant improvement was when using Transformers as a decoder when comparing different combinations of RNNs and Transformers as decoders and encoders. A possible interpretation of this result is that Transformer architecture better utilizes the metadata, i.e., the collision energy and charge state information. A future direction of the project could be to investigate the source of the improved accuracy by examining the effects of removing this information from the different decoders.

Here, we made use of the framework provided by the original Prosit project. It was essential to access the scripts and data sets provided and hardened by the previous team of algorithm designers. In general, it is of utmost importance to keep this type of resource easy to access. If we want to attract the attention of the machine learning community, which often wants a precise problem formulation and does not like to get into the details of how to generate data sets from scratch, we need to help them.
